# A qualitative exploration of discharge destination as an outcome or a driver of acute stroke care

**DOI:** 10.1186/1472-6963-14-193

**Published:** 2014-04-29

**Authors:** Julie A Luker, Julie Bernhardt, Karen A Grimmer, Ian Edwards

**Affiliations:** 1 International Centre for Allied Health Evidence, University of South Australia, GPO Box 2471, 5000 Adelaide, South Australia; 2 Florey Institute of Neurosciences & Mental Health, University of Melbourne, NHMRC Research Fellow, Melbourne, Victoria, Australia

**Keywords:** Quality of care, Stroke, Decision making, Evidence-based practice, Health care disparities, Allied health professionals

## Abstract

**Background:**

Many patients with acute stroke do not receive recommended care in tertiary hospital settings. Allied health professionals have important roles within multidisciplinary stroke teams and influence the quality of care patients receive. Studies examining the role of allied health professionals in acute stroke management are scarce, and very little is known about the clinical decision making of these stroke clinicians. In this study we aimed to describe factors that influence the complex clinical decision making of these professionals as they prioritise acute stroke patients for recommended care. This qualitative study was part of a larger mixed methods study.

**Methods:**

The qualitative methodology applied was a constructivist grounded theory approach.

Fifteen allied health professionals working with acute stroke patients at three metropolitan tertiary care hospitals in South Australia were purposively sampled.

Semi-structured interviews were conducted face to face using a question guide, and digital recording. Interviews were transcribed and analysed by two researchers using rigorous grounded theory processes.

**Results:**

Our analysis highlighted ‘predicted discharge destination’ as a powerful driver of care decisions and clinical prioritisation for this professional group. We found that complex clinical decision making to predict discharge destination required professionals to concurrently consider patient’s pre-stroke status, the nature and severity of their stroke, the course of their recovery and multiple factors from within the healthcare system. The consequences of these decisions had potentially profound consequences for patients and sometimes led to professionals experiencing considerable uncertainty and stress.

**Conclusions:**

Our qualitative enquiry provided new insights into the way allied health professionals make important clinical decisions for patients with acute stroke. This is the first known study to demonstrate that the subjective prediction of discharge destination made early in an acute admission by allied health professionals, has a powerful influence over the care and rehabilitation provided, and the ultimate outcomes for stroke patients.

## Background

Despite strong evidence to guide best-practice care for all stroke patients, many patients do not receive recommended care [1,2]. Less than optimal care can lead to poor stroke outcomes [3,4]. It is therefore essential to understand why care varies so that clinical quality improvement initiatives can be effectively targeted.

The predominant research on the quality of care provided to patients with acute stroke has used quantitative methodologies to explore medical interventions or healthcare systems. Quantitative studies have linked the quality of care received by patients with acute stroke to predictor variables such as age [5-7] and day of hospital admission [8]. As part of a larger mixed methods study, we have also published quantitative research that investigated associations between the quality of care acute stroke patients receive from AH professionals and various predictor variables including age, gender, stroke severity, Charlson Comorbidity Index, English proficiency, previous accommodation, previous independence level [2,9].

There is a growing body of literature that acknowledges the influence of the organisational structures or systems on the quality of stroke care and patient outcomes. Patients’ with acute stroke achieve the best outcomes if cared for in stroke units where coordinated team based care is provided by professionals with specialised skills [3]. In addition to the influence of organisational structures, the processes of care that patients receive is a consequence of the multiple clinical decisions made by members of the clinical team. There is a current lack of understanding of how and why stroke clinicians, including allied health (AH) professionals, make decisions regarding clinical care.

The Australian Clinical Guidelines for Stroke Management [4] state that AH professionals are key members of stroke teams and these teams usually include physiotherapists, occupational therapists, social workers, speech pathologists, dietitians and psychologists. Their roles include the clinical assessment of the consequences of stroke, the provision of early therapy and rehabilitation, the prevention and management of complications, and discharge planning. Many decisions in stroke care, such as patients’ discharge destination and suitability for rehabilitation, are not made on purely medical grounds. AH professionals have a key role in determining patients’ safety and rehabilitation potential, and are strongly positioned to influence patients’ outcomes. Studies examining the role of AH professionals in acute stroke management are scarce, and very little is known about the clinical decision making of these stroke clinicians.

Qualitative research methodology is most appropriate for understanding complex constructs such as decision making [10]. To date however, there has been very little qualitative exploration of the underlying drivers of decisions regarding any care that stroke patients will, or will not receive [11].

In this qualitative enquiry, our aim was to explore how and why AH professionals make decisions about clinical prioritisation and the processes of care they provide, or don’t provide, to patients with acute stroke. We sought to incorporate their voices to help describe the meaning or intention behind different care decisions and the types of care being provided.

## Methods

### Our mixed methods framework

This article will focus on the qualitative enquiry within our larger mixed methods study. Mixed-methods research allows a more complete understanding of acute stroke care though the integration of both quantitative and qualitative methods [12]. We conceived a mixed methods study to explore the factors influencing the quality of care provided to patients with acute stroke by AH professionals.

The four largest tertiary hospitals that admitted acute stroke patients in Adelaide, South Australia were approached to participate. One site was subsequently excluded as it was undergoing organisational changes that would have confounded the research. At the time of the study the three participating hospitals admitted between 180 – 450 acute stroke patients per year.

Details of the quantitative study are published elsewhere [2,9]. In summary the quality of care provided by AH professionals for consecutively admitted acute stroke patients was determined by the adherence of care to 20 predetermined evidence-based AH process indicators. The retrospectively audit of 300 patients at the three participating hospitals (100 per hospital), found poor adherence to recommended care for most patients (see Additional file 1). A concurrent qualitative study (reported here) then explored this issue further. In the larger study, a final step of mixed methods analysis merged and compared the results from the two methodologies [13]. The side-by-side comparison technique described by Creswell & Plano Clark was used to undertake the final mixed methods analysis [14].

In this study we chose a constructivist grounded theory approach which is recommended when examining poorly understood phenomenon [15]. Using this methodology we explored the perceptions of AH professionals regarding their decision making and the prioritisation of acute stroke patients to receive various assessments, rehabilitation therapy and other elements of recommended care. Ethical approval was obtained from the University of South Australia’s Human Research Ethics Committee, the Southern Adelaide Clinical Human Research Ethics Committee and the Central Northern Adelaide Health Service Ethics of Human Research Committee.

### Study sample

AH professionals at the three audited hospitals in South Australia were invited to provide informed consent to participate if they had worked with acute stroke patients at their hospital for at least six months. Interviews were conducted during 2010 at a convenient time and place for consenting participants. Following purposive sampling principles, we recruited a mix of qualified physiotherapists (PT), speech pathologists (SP), occupational therapists (OT), social workers (SW), dietitians (DN) and psychologists (PS) across all three sites. No more than two representatives from the same AH discipline were recruited from a particular site.

We recruited 15 allied health professionals, five from each case study site. We conducted interviews with five PTs, three SPs, three DNs, two OTs and two SWs. Interviewees had various levels of experience and seniority within their hospitals, ranging from inexperienced first year graduates to very experienced professionals with over 20 years’ work in stroke clinical and leadership roles. We were reassured that thematic saturation had been reached for this sample of AH professionals, as no new concepts emerged during the last three interviews. Thematic saturation was not assessed for discipline subsets.

### Data collection & management

Using an interview guide we conducted semi-structured interviews which allowed for probing or additional questions as interesting concepts emerged (see Table 1). These guiding questions were informed by an extensive literature review [16], the results of our earlier quantitative study [2,9] and the interviewing framework suggested by Charmaz [15]. Interviews were audio recorded and transcribed verbatim. We returned the transcripts to participants for member checking to allow them to clarify their intended meaning if required [15,17]. Throughout the interviews, field notes were taken to allow us to add context and richness to the data collected and to facilitate the tracking of our thinking and decision making processes [18]. Prior to analysis, interviewees and hospitals were de-identified. Unfortunately equipment failure led to the loss of one digitally recorded interview so that field notes only were analysed for this participant.

**Table 1:** Interview question guide

	**Interview question guide regarding stroke management**
1	What do you like and not like about working with stroke patients in this hospital?
2	Tell me about your work with stroke patients who at this hospital.
3	How are clinical decisions made in your hospital, regarding the clinical management of individual patients?
4	How do you prioritise your clinical work?
5	What do you consider when deciding on a patient’s potential to benefit from rehabilitation?
6	Are there any systems or tools available to you in this workplace to help you with prioritisation of your clinical workloads?
7	How confident do you feel in your knowledge of acute stroke management?
8	Are there other issues that influence your work with acute stroke patients?

NVivo 8 computer software assisted us with data management [19].

### Analysis

**Figure 1:**
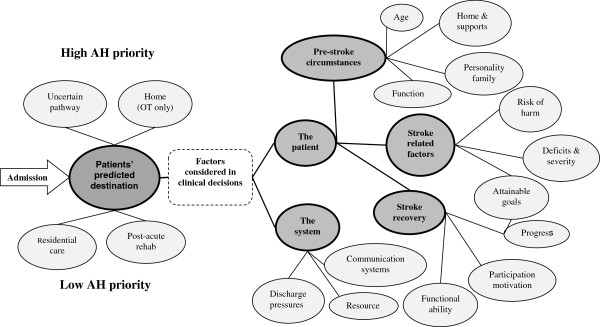
Concept map of themes emerging from the data.

Analysis was undertaken in line with Charmaz’s constructivist grounded theory approach [15]. We developed familiarity with the data through repeated listening and reading of the interviews. The transcripts were inductively coded by two independent researchers (JL & IE), according to the grounded theory processes of open or initial coding, focused coding, axial coding and theoretical coding [15,20]. We commenced open coding during the data collection period [18]. This enabled early analysis and preliminary thematic interpretation to inform our later interviews if required. This initial coding stayed close to the data, by coding directly onto transcript margins and using in-vivo codes and direct quotations [15]. Through a collaborative approach with regular meetings, we added depth to our understanding, established consensus and developed focused coding of the emerging concepts. Common themes emerged regarding AH professionals’ clinical decision making and patient prioritisation. This iterative process of developing emergent themes was captured in memos, using NVivo software and also represented figuratively in concept maps (see example Figure 1) [21,22]. Through this constant comparison we sought relationships between themes to form the basis of emerging assertions and theories regarding issues of relevance to the research focus [15,23].

## Results

The main emergent themes were *prediction of discharge destination, pre-stroke status, the stroke, stroke recovery, the system,* and *deserving a chance,* each of which had several sub-themes*.* The interviewed AH professionals from all disciplines described the need to make complex decisions about the priority of individual patients to receive specific recommended processes of care.

The prediction of discharge destination emerged as a dominant and unexpected driver of decisions for AH professionals regarding the care patients would receive. This concept of discharge prediction had important implications for patients and required staff to consider factors that fell broadly into four categories: the patient’s status before their stroke, the nature of their stroke, their course of stroke recovery and organisational factors within the health system.

In a fast paced, multi-disciplinary clinical environment, where the organisational priority was to maintain a smooth, swift flow of patients out of the acute setting, interviewees conveyed a desire to give patients ‘every chance’ to make an optimal stroke recovery. This complex interplay of factors caused uncertainty for AH professionals and in some cases distress.

### Prediction of discharge destination

The prediction of discharge destination pre-empted decisions about much of the care patients would receive. Early in admission AH professionals aimed to determine patients’ ultimate discharge destination through complex clinical reasoning. One SP described her thought processes as:

Thinking about what’s the ultimate goal . . . Is the aim to get them home? Is the aim to improve their function to, perhaps instead of going into [nursing home] care, going into [hostel] level care?

When this decision was made it governed the patient’s clinical priority and the amount and type of care they would receive. A DN explained the importance of this decision:

You need to have that clinical understanding of what’s likely to happen with a patient with a certain type of stroke…So if you’ve got an idea of where they are going you can adapt your plans and interventions more specially to those patients I guess.

Patients became a low priority for AH care if they were thought to be destined for residential care placement, or were on a palliative care pathway.

Once patients were thought suitable for discharge directly to home, they tended to become a lower priority for all disciplines except the OTs who played a key role in home set-up. Interviewees spoke with regret of the need to also give low priority to patients who had been accepted for a post-acute rehabilitation bed. One OT put it this way:

Well once people are accepted for rehab that becomes then a [low] priority. Unfortunately, in this environment, therapy is lower priority, because we’re having to be reactive to all the discharge issues in this environment. Unfortunately.

A PT affirmed this:

*When we are really busy and we have to drop people off the list, the nursing home assessed patients get dropped off the list first. People who we know are going to go to rehab and have already been accepted are dropped off the list as well which is unfortunate*.

The consequence of this was that patients deemed to need stroke rehabilitation were no longer likely to receive that care in the acute setting and so important therapy would be delayed until a bed was available in a post-acute centre.

AH professionals reported that patients who had an unclear discharge pathway were a particularly high priority for their attention. Interviewees spoke of providing additional care and consideration to patients with undetermined discharge destinations, to enable the gathering of new information required for decision making. One SP described this as “diagnostic rehab therapy”, which assisted her to “demonstrate that they can show improvement and get them to the right place.” A PT clearly identified the priority to clarify discharge pathways, in this way:

So our first sort of consideration would be in the morning, the new patients and the ones that don’t have clear sort of pathways that they’re on . . . and being able to determine whether or not they are rehab or whether or not they are not rehab.

There was awareness that patients with unclear discharge destinations presented a risk to the smooth flow of patients through the acute and post-acute hospital systems. One PT said:

The ideal patients maybe from the system’s point of view that will move through the system quicker and get back to their home rather than getting stuck in a bed.

Another OT demonstrated the covert nature of this imperative when she said:

I think that whilst it may not be in writing, I think that it’s not always looked on as favourably if the patient doesn’t have a discharge destination.

#### Difficulties in predicting destination

AH professionals considered ‘predicted discharge destination’ in a manner that had the potential to significantly influence the quality of care and the clinical outcomes for patients. The prediction of stroke recovery was described by an experienced SP as challenging and a skill that required considerable clinical experience:

The challenge is often that we see them when they are at their most severe …I think if you have only ever worked with stroke patients in the super-acute setting you don’t realise how well they do further down the track and what the signs are of who’s going to do better or worse despite the severity of their disability.

Several less experienced AH professionals described acute stroke work as very demanding and that it took time and additional training to develop the necessary skills. One PT reported the surprise of encountering a patient who, against the odds, made late, unexpected improvements just in time for it to be considered in destination decisions.

This illustrated the risks of using destination as a driver of care decisions for this patient group in a fast-paced environment where important judgments are made early in admission. The decision making regarding destination was influenced by a complex interplay of factors related to the patient, their stroke and also health system variables as discussed below.

### Considering pre-stroke status

When clinical care decisions were made, including the prediction of discharge destination, factors associated with a patient’s health and lifestyle prior to their stroke were considered.

#### Patients’ age

Many AH professionals reported that a patient’s age influenced the quality of care they would receive. Interviewees spoke of a greater momentum within the clinical team to provide optimal care for younger patients who had suffered a stroke, than was seen for older patients. Several AH professionals described a greater sense of loss for young stroke survivors who might have ongoing commitments such as employment and a young family. Concerns were raised in several interviews that older patients could be unfairly denied access to recommended care. An example from one PT:

I guess you hear even rehab doctors talking about it all the time, ‘We better give that person in their 40s every chance’. But you don’t hear them talking about that with someone in their 80s.

While some interviewees spoke of ageist inequities in care others argued that it was the factors that could accompany older age, or age proxies, which were the true drivers of care decisions.

*Actually I don’t think age* per se*. I think level of function prior to admission is actually more important. . . . And when I am thinking of rehab it is thinking about, you know, what were they like, what will they be going back to, not about age* per se*,* reported one SP.

A SW clearly expressed her non-ageist views:

I think that the human condition is probably more significant than any age factor and there may be older people who are.. that might really have their act together extremely well…

#### Previous functional independence, accommodation and supports

Patients were considered good candidates for rehabilitation, or for discharge directly home, if they had been previously independent and if they had adequate family supports available. A previously proven physical capacity to undertake the rigors of intensive therapy was also deemed necessary before referring to post-acute rehabilitation.

#### Personality and culture

Interviewees acknowledged that the personality and assertiveness of patients and their families occasionally had an influence on care decisions. Where patients or families were particularly anxious or demanding of more therapy, this was sometimes provided even if not considered to be a clinical priority. Although English as a second language ESL was thought to delay care on occasions when interpreters were required, no one reported that patient’s priority for care was influenced by their cultural background.

### Considering the stroke

The prediction of discharge destination also required consideration of the type of stroke sustained and the course of recovery.

#### Stroke deficits and risks

Certain stroke-related deficits placed patients at risk of further deterioration. A common example was swallowing disorders which increased the risks of aspiration pneumonia and malnutrition for patients and therefore made these patients a high priority for attention from SPs and DNs. Although predicted discharge destination appeared to determine the clinical priority for most stroke patients, these very high risk patients were an exception and were a priority for AH care irrespective of their discharge path. Certain stroke-related deficits, such as severe cognitive impairments, also influenced the ability of patients to receive recommended care.

#### Stroke severity

Several AH professionals reported enjoying the challenge and rewards of working with severely affected stroke patients despite the hard physical work and discharge difficulties these patients presented. From one PT:

Those patients who are more reliant on your help in rehabilitation have more priority with me. I find that a lot more rewarding.

However despite the extra effort afforded to severely affected patients, it was recognised that the patients with milder stroke deficits were more likely to be receive evidence-based rehabilitation. In another PT’s words

They [post-acute rehabilitation hospital] tend to take those patients that are maybe going to get back to almost the way they were previously pre-stroke.

### Considering stroke recovery

The perception of staff regarding the course of stroke recovery in the acute setting was a key consideration for important clinical decisions, such as patients’ suitability for post-acute rehabilitation.

#### Progress

It was important for patients to demonstrate progress in stroke recovery and AH professionals saw that an important part of their role was to facilitate this demonstration of progress.

*Before I refer them to rehab we like to know that they are making gains, even if they are small gains* reported one PT.

#### Participation, motivation & depression

Patient’s capacity or willingness to actively take part in therapy sessions affected their priority for AH professionals’ attention.

*. . . my higher priority would be to see patients who are participating well,* explained one PT. Poor participation was a barrier to successful referral for further rehabilitation or for an optimal discharge destination, as expressed by one SW:

A big thing is if they are participating with the acute OTs and physios, because if they are not participating with them it is highly unlikely that they will participate with [post-acute rehab].

Many interviewees reflected on the role of motivation in patients’ stroke recovery and the care that they received. Patients who were unmotivated to participate in therapy sessions were difficult and often unrewarding for AH professionals. Several interviewees reported that it was an important part of their role to try to motivate patients by using strategies such as building rapport or developing patient-centred goals, while others felt it was beyond their influence.

Depression was raised as a complicating factor after stroke and a potential barrier to optimal care. Examples were cited where patients had only started to engage effectively in therapy sessions and make functional improvements after commencing anti-depressant medication. The pressure for fast discharge from the acute setting often meant that depressed patients did not have time for medication to take effect and then to demonstrate recovery potential from their stroke. One SP lamented:

If [depression] is not recognised and that is not treated then often people will be written off as not engaging in rehab and diagnostic therapy, when in fact it is depression.

The complex interplay of variables related to an individual patient’s clinical presentation was further complicated by the influence of issues stemming from the health system.

### Considering the system

The system in which AH professionals worked had a reported influence on the quality of care that they provided.

#### Structural resources

Unsurprisingly some systematic factors varied across hospital sites due to structural differences such as the availability of stroke unit beds. Poor AH staff-patient ratios in the acute setting were universally reported to be a barrier to providing recommended care. Inadequate post-acute inpatient rehabilitation beds influenced AH professionals’ prioritisation of patients to receive, or not receive, recommended care. Some interviewees expressed a responsibility to use scarce resources wisely, as demonstrated by this PT:

..there is only a limited place for rehab beds in the state and that’s a big factor to decision making. You are sitting there [at discharge planning meetings] thinking in your head that those beds should be used well in a way if that makes sense.

#### Discharge pressures

The pressure to move patients rapidly out of the acute hospital was considered to be a significant influence on the care patients received. Hospital priority systems were in place to manage the risk of low bed capacity and this demanded that all AH professionals made patients’ discharge a clinical priority. The overpowering effect of discharge pressures strongly influenced the entire clinical priority and decision making process for all AH professionals. There was awareness that patients with unclear discharge destinations presented a risk to the smooth flow of patients through the acute and post-acute hospital systems, and justified making them a clinical priority.

#### Communication systems

Communication systems, team work and a hierarchy of decision makers were raised as influences on stroke care. Where referral systems were poorly developed, patients’ care could be severely compromised by long delays before being seen by AH. The system of referring patients on to post-acute rehabilitation facilities was universally seen as problematic. The assessment criteria for acceptance to rehabilitation were considered to be non-transparent, subjective and *“some of the rehab facilities would change the goal posts”* (OT)*.* Decisions around the appropriateness and timing of referrals to post-acute rehabilitation facilities were important but difficult tasks for AH professionals:

*We constantly negotiate with each other … it is that fine line, if you put it [referral to post-acute rehab] in too soon you will get knocked back, if you put it in too late they are hanging around,* reported one PT.

As detailed above and in the concept map (Figure 1), interviewees described a process of complex clinical decision making in which multiple factors needed to be considered concurrently, and frequently resulted in AH professionals experiencing uncertainty and stress.

### Deserving a chance

AH professionals conveyed a strong sense of responsibility for the outcomes of their patients and an ethical sense that *“everybody probably deserves a chance”*(PT). There was a high level of awareness of the evidence-based recommendations for stroke management. This was offset by a clear recognition that some patients had greater difficulty securing recommended care such as post-acute rehabilitation. Patients considered to be at-risk included those with more severe strokes, older age, lower levels of home support, cognitive deficits and poor motivation or depression. The challenge of securing rehabilitation opportunities for at-risk patients placed pressure on acute AH staff to advocate for these patients and demonstrate a potential to make functional improvements.

Some interviewees communicated great insight into the difficulties they faced when attempting to provide equitable care. This ethical strain was most frequently raised when discussing the influence patients’ age had on care decisions as demonstrated by an OT:

I think I tend to spend a lot more time on the younger demographic . . . and I’m not sure how comfortably that sits with me now, now that I’ve actually mentioned it.

There was frequently a tension between what interviewees knew to be evidence-based care and the care that they could actually deliver. High patient caseloads caused a level of distress for some staff as they attempted to use scarce resources wisely while providing quality care for their patients. In the words of an OT:

I don’t enjoy the lack of funding, resources, man power in the acute setting and the feeling that I wish I could do more for patients, and I’m not always able to because of those restrictions . . . So I feel a bit torn sometimes by that, because I don’t like going away feeling like I haven’t done my best, but I’ve done what I could in the time available.

## Discussion

The main theoretical contribution made by our study was the emergence of ‘predicted discharge destination’ as a major driver of care decisions for this professional group. This new understanding of discharge destination as a determinant of care is quite different from our usual consideration of discharge as an outcome of care. The determination of a discharge destination was a priority task for AH professionals, and patients with unclear discharge pathways were given precedence for attention. Once a discharge decision was made for a patient it had an overriding influence on their priority to receive processes of care from AH professionals. This had major potential consequences for many patients. Those thought to be destined for discharge directly home or to post-acute rehabilitation centres were unlikely to receive any further therapy in the acute setting, and patients waiting for discharge to a residential care bed became a very low priority for AH care. Only those patients deemed to be at high risk of deterioration, such as those with stroke-related swallowing problems, remained a high priority for AH irrespective of their discharge pathway.

### Decision making conundrums

Our interviewees reported the regular employment of high level, complex clinical decision making. Recent research reported that well developed clinical decision making skills require access to a variety of clinical experiences and sufficient time for skill development [24]. Participants in our study, with varying levels of experience, reported that clinical experience was particularly important in the specialised field of acute stroke management. In many settings the reality is that novice AH professionals are placed on short roster rotations to work with acute stroke patients. These novice staff might not have the opportunity to develop the necessary level of clinical problem solving skills prior to making complex decisions, which could then have profound consequences for patients.

The power of “predicted destination” to drive care decisions and clinical priority presents particular problems for the quality of care received by patients with acute stroke. Stroke recovery is often unpredictable and complicated by the inability of some patients to demonstrate their potential to make recovery during the early days after stroke onset [25,26]. In post-acute rehabilitation settings the difficulties of team decision making for stroke patients’ discharge destination have been investigated and researchers have recommended the use of objective models to assist consistency and transparency [27]. In acute settings, where there is increased pressure to discharge patients quickly, mistakes can be made if important decisions are made too soon and might result in some patients missing out on opportunities to maximise their recovery.

### Ethical implications for AH professionals

AH professionals worked with high levels of clinical uncertainty within a fast paced health system driven by pressures to discharge quickly. Our study revealed a tension between what AH professionals knew to be best practice or ethically correct, and what they could actually provide for their patients. Staff were left to rationalize and manage the resulting distress sometimes experienced.

### Limitations of our study

Our findings are limited by a relatively small sample size. We are reassured by the depth and richness of interview data we gathered and our determination that data saturation had been achieved. Data collection from several AH disciplines at multiple hospitals added credibility to our findings. Our study sought to understand the way AH professionals make care decisions for acute stroke patients. We acknowledge that these clinicians are components of larger stroke clinical teams, and that the decision making of nurses, doctors and other professions also impact of patient care. We also acknowledge that our study was conducted in one Australian city and therefore findings might not fully transfer internationally where health systems will vary.

### Next steps

The discovery of ‘predicted discharge’ as a driver of care quality provides important new information for future clinical quality stroke research. Discharge destination is usually considered to be an outcome of inpatient care rather than a causal factor for the quality of care patients receive. Previous quantitative enquiry has therefore not factored discharge destination into statistical modelling when investigating the determinants of care quality. Future research can now test whether discharge destination, or its prediction, is supported statistically as well as qualitatively as a driver of care.

## Conclusions

Allied health professionals working in acute hospital settings, undertook complex decision making when deciding on the processes of recommended care that patients would or would not receive. Systematic pressures from low allied health resourcing and the need for fast discharge meant that patients were prioritised for allied health interventions. This prioritisation resulted in some patients missing out on optimal care with possible negative consequences for their ultimate stroke outcomes. Prioritisation for care was based on allied health professional’s subjective prediction of the patients’ discharge destination, which in turn was founded on multiple factors, many of which were unquantifiable.

The desire to provide fair and unbiased, high quality care for patients drives many initiatives in clinical quality improvement. Given the complexity of clinical decision making for acute stroke patients however, more qualitative and quantitative exploration is required before equitable systems of care delivery will result.

## Competing interests

No competing interests are declared.

## Authors’ contributions

All authors contributed to the study design and manuscript editing. JL conceived of the study, performed the interviews, collected, coded and interpreted the data, and drafted the manuscript. JB and KG provided content expertise and critical evaluation. IE advised on the methodology and analysis, participated in data coding and interpretation and provided critical evaluation. All authors read and approved the final manuscript.

## Pre-publication history

The pre-publication history for this paper can be accessed here:

http://www.biomedcentral.com/1472-6963/14/193/prepub

## Supplementary Material

Additional file 1 Results of clinical audit of care provided to 300 patients. Click here for file
