# Supplementary material for: A qualitative exploration of discharge destination as an outcome or a driver of acute stroke care
Source: BMC Health Serv Res. 2014 Apr 29;14:193. doi: 10.1186/1472-6963-14-193 (PMC4045916; doi:10.1186/1472-6963-14-193)
Supplement: Additional file 1 — Results of clinical audit of care provided to 300 patients. [file 1472-6963-14-193-S1.docx]

# Additional files

### Additional file 1 – Results of clinical audit of care provided to 300 patients

| **Process indicator** | **Patients**  **Eligible for Process Indicator** | | **Process Indicator Adherence for those Eligible** | |
| --- | --- | --- | --- | --- |
|  | N= | % | N= | % |
| 1. Swallow screening | 279 | (93) | 144 | (51.6) |
| 1. PT assessment   (<48 hours of admission) | 275 | (91.7) | 167 | (60.7) |
| 1. OT assessment   (<48 hours of admission) | 275 | (91.7) | 97 | (35.3) |
| 1. SP assessment   (<48 hours of admission) | 275 | (91.7) | 174 | (63.3) |
| 1. DN assessment   (<48 hours of admission) | 274 | (91.3) | 24 | (8.8) |
| 1. SW assessment   (<7 days of admission) | 277 | (92.3) | 71 | (25.6) |
| 1. Cognitive assessment | 244 | (81.3) | 47 | (19.3) |
| 1. First mobilisation <24 hours of stroke onset | 211 | (70.3) | 25 | (11.8) |
| 1. Early PT rehabilitation (<48 hours of admission) | 195 | (65.0) | 20 | (10.2) |
| 1. Early SP rehabilitation (<48 hours of admission) | 168 | (56.0) | 2 | (1.2) |
| 1. Early OT rehabilitation (<48 hours of admission) | 205 | (68.3) | 3 | (1.5) |
| 1. NGT process | 42 | (14.0) | 41 | (97.6) |
| 1. Nutritional screen | 193 | (64.3) | 0 | (0) |
| 1. Mood assessment | 224 | (74.6) | 1 | (0.5) |
| 1. Family meeting | 292 | (97.3) | 34 | (11.6) |
| 1. Stroke education | 273 | (91.0) | *33 | (12.1) |
| 1. Secondary stroke prevention | 207 | (69.0) | 17 | (8.2) |
| 1. Home visit | 49 | (16.3) | 2 | (5.0) |
| 1. Carer skills training | 39 | (13.0) | 1 | (2.6) |
| 1. Discharge plan provided | 131 | (43.7) | 12 | (9.2) |
| **Legend:** PT= Physiotherapy OT= Occupational therapy SP= Speech pathology  SW= social work DN= Dietetics * = missing data for one patient  **Definitions:** First mobilisation= documentation of patient first sitting out of bed or ambulating with or without assistance.  Rehabilitation= therapeutic interventions to retrain neurological deficits or teach compensatory techniques aimed at improving function (excludes assessment).  NGT process= Naso-gastric tube feeding if no functional swallow during the first month post stroke  Home visit= environmental assessment by OT or PT to facilitate discharge to home (with/without patient present) | | | | |
